# Fasting Serum IGFBP-1 as a Marker of Insulin Resistance in Diverse School Age Groups

**DOI:** 10.3389/fendo.2022.840361

**Published:** 2022-05-02

**Authors:** Amrit Bhangoo, Rishi Gupta, Steve P. Shelov, Dennis E. Carey, Siham Accacha, Ilene Fennoy, Lisa Altshuler, Barbara Lowell, Robert Rapaport, Warren Rosenfeld, Phyllis W. Speiser, Svetlana Ten, Michael Rosenbaum

**Affiliations:** ^1^Division of Pediatric Endocrinology, Children’s Hospital of Orange County, Orange, CA, United States; ^2^Department of Pediatrics, Division of Pediatric Gastroenterology and Endocrinology, University of Rochester Medical Center, Rochester, NY, United States; ^3^Department of Pediatrics, Winthrop University Hospital, Mineola, NY, United States; ^4^Division of Pediatric Endocrinology, Northwell Health, Lake Success, NY, United States; ^5^Division of Pediatric Endocrinology, New York Presbyterian Morgan Stanley Children’s Hospital, New York, NY, United States; ^6^Program for Medical Education Innovations & Research (PrMeir), New York University (NYU) Grossman School of Medicine, New York, NY, United States; ^7^Laboratory of Diabetes, Obesity and Other Metabolic Disorders, Feinstein Institutes for Medical Research, Northwell Health, Manhasset, NY, United States; ^8^Division of Pediatric Endocrinology and Diabetes at Mount Sinai Kravis Children’s Hospital, New York, NY, United States; ^9^Cohen Children’s Medical Center of NY and Zucker School of Medicine, New Hyde Park, NY, United States; ^10^Division of Pediatric Endocrinology, Richmond University Medical Center, Staten Island, NY, United States; ^11^Department of Pediatrics, Division of Molecular Genetics, New York Presbyterian Medical Center, New York, NY, United States

**Keywords:** IGFBP-1, adiposity, insulin resistance, BMI – body mass index, waist circumference

## Abstract

**Introduction:**

The known markers of insulin resistance in obese children are well studied. However, they require serial measurements and complicated calculations. The objective is to study IGFBP-1 and its relation with other known risk measures.

**Materials and Methods:**

The study included 98 New York City school students of diverse ethnic/racial backgrounds (57 males and 41 females), 11–15 years of age. Subjects were enrolled in a cross-sectional study, and anthropometric measures were collected. They underwent fasting intravenous glucose tolerance tests (IVGTT), and glucose, insulin, lipids, IGFBP-1, adiponectin and inflammatory markers were collected.

**Results:**

The subjects were stratified into 3 groups based upon the BMI Z-score. Out of all the subjects, 65.3% were in the group with a BMI Z-score <1 SDS, 16.3% subjects were in the group with a BMI Z-score of 1 to 2 SDS, and 18.4% of the subjects were in the group with a BMI Z-score of more than 2 SDS. The group with a BMI Z-score of more than 2 SDS had increased waist circumference (WC), body fat, increased fasting insulin, and triglycerides (TG). This group had decreased levels of adiponectin and HDL and low IGFBP-1 as compared to the group with BMI <1 SDS. The group with a BMI Z-score of 1 to 2 SDS had a decreased level of IGFBP-1 as compared to the group with a BMI Z-score less than 1 SDS. IGFBP-1 inversely correlated with age, WC, BMI, body fat, TG, and insulin levels. IGFBP-1 positively correlated with adiponectin and HDL levels.

**Conclusion:**

IGFBP-1 in children can identify the presence of insulin resistance in the group with BMI 1 to 2 SDS, even before the known markers of insulin resistance such as elevated triglycerides and even before decreased HDL and adiponectin levels are identified.

## Introduction

The prevalence of obesity among US children was recorded to be at 18.5% as per the National Survey of Children’s Health (NSCH) in 2016 versus 10% in the 2003 survey (1). Total body adiposity, especially abdominal fat, increases risk factors for type 2 diabetes, insulin resistance, cardiovascular disease, dyslipidemia, non-alcoholic steatohepatitis (NASH), and circulating concentrations of pro-inflammatory cytokines (1–8).

Fasting hyperinsulinemia is associated with insulin resistance (9, 10), but intravenous and oral glucose tolerance tests (IVGTT and OGTT) predict better insulin resistance in obese children despite having similar fasting serum insulin levels (11–13). A number of indices of insulin sensitivity such as fasting insulin, quantitative insulin sensitivity check index (QUICKI), homeostatic model assessment for insulin resistance (HOMA), acute insulin response (AIR), and glucose disposal index (GDI) have variable correlations with the so-called gold standard of a euglycemic clamp study and with body fatness. Minimal model IVGTT (a 180-min test with frequent blood sampling) and euglycemic–hyperinsulinemic clamps are a more accurate method of quantifying insulin sensitivity (Si), GDI, and AIR (14, 15). Euglycemic clamp studies, though considered the gold standard in measuring insulin sensitivity, are impractical in a pediatrician’s office as they required serial measures during several hours and require complicated calculations of insulin sensitivity indices (16). There is a need to identify an early, convenient, and simple marker for assessing insulin resistance in children instead of serial laboratory values and complex formulas.

Insulin-like growth factor binding protein-1 (IGFBP-1) is a 28-kD protein, mainly produced in the liver, and its secretion is inhibited by insulin (17, 18). IGFBP-1 was shown to be useful in the assessment of whole-body insulin sensitivity and is an adiposity-independent liver-specific circulating marker of insulin sensitivity in adults and adolescents (4, 19–21). IGFBP-1 may constitute as an independent risk factor for the development of insulin resistance, increased risk of type 2 diabetes, and other adiposity-related comorbidities in children, even at a young age. Circulating concentrations of IGFBP-1, coupled with fasting levels of insulin, have been proposed as a more specific screening test for insulin resistance in children (5, 22, 23). We propose that IGFBP-1 should be considered as marker of insulin resistance in children, and it is easier to obtain than other commonly used indices of insulin sensitivity.

## Materials and Methods

### Subjects

As a part of this cross-sectional study, fasting blood samples were obtained from 98 middle school subjects (age 11–14 years, mean age 12.5 years; 57 males, 41 females). All willing subjects were consented and enrolled in the ROAD (Reduce Obesity and Diabetes) Project. The ROAD Project was a multisite 5-year study of a multiethnic population of middle-school students in 5 New York City public schools implemented by a collaborative research consortium of academic medical centers called the ROAD Study. The project had been coordinated through the Academy for Medical Development and Collaboration (AMDeC) New York, NY (7). The collaborating institutions were Cohen Children’s Medical Center, Columbia University Medical Center, Maimonides Medical Center, Mt. Sinai Medical Center, and Winthrop University Hospital.

**Figure 1:**
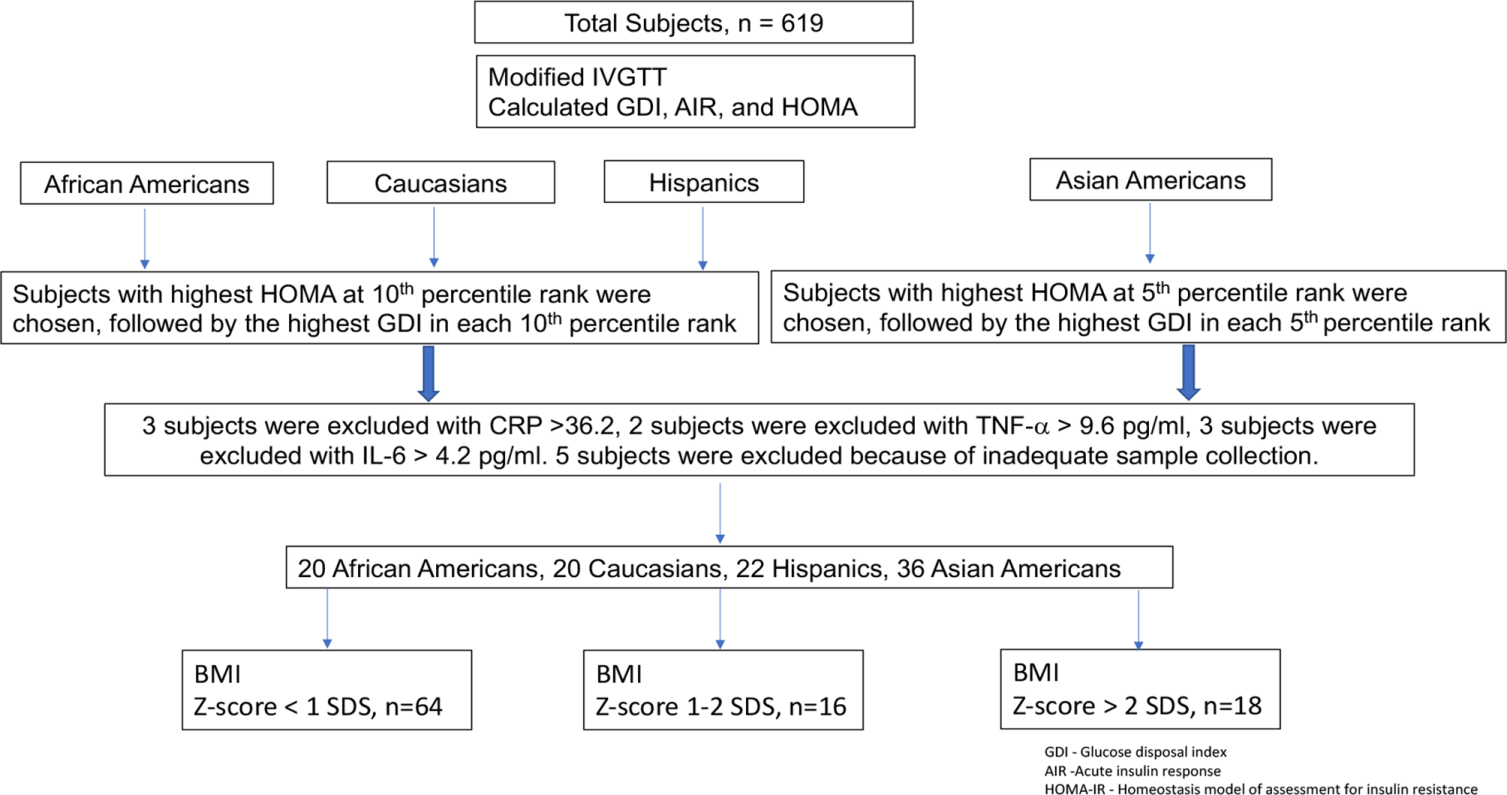
Study selection outline of the study participants, testing procedures, and subject selection.

An initial population of 619 subjects was divided into 4 groups based on self-identification of ethnicity/race: African American, Caucasian, Hispanic, and Asian American. For this substudy, subjects were selected from initial screening tests performed in the fall assessment session of the first year they were enrolled. Within each ethnic/racial group, subjects were ranked from the lowest to highest values for measures of insulin secretion and resistance: glucose disposal index (GDI), acute insulin response (AIR), and homeostasis model of assessment for insulin resistance (HOMA) (24). Subjects in the African American, Caucasian, and Hispanic groups with the highest HOMA in each 10th percentile rank were chosen, followed by the highest glucose disposal index in each 10th percentile rank to generate a total of 22–25 subjects per group. In the case of Asian-Americans, a similar procedure was used for the highest values of HOMA and GDI in each 5th percentile rank to generate a total of 38 subjects to include a mixed population of East Asian (from China, Japan, Taiwan, and Korea) and South Asian (from India, Pakistan, Sri Lanka, Bangladesh) subjects. Outliers for fasting insulin or inflammatory markers—tumor necrosis factor-α (TNF-α), adiponectin, C-reactive protein (CRP), interleukin-6 (IL-6)—more than 3 SDS from the mean were eliminated prospectively on the assumption that such subjects probably either were not truly fasting or were ill. More specifically, 5 subjects were excluded for insulin >40 mIU/ml; 3 subjects were excluded because of IL-6 >4.2 pg/ml; 2 subjects were excluded because of TNF-α >9.6 pg/ml; and 3 subjects were excluded because of CRP >36.2 pg/ml. There was some overlap between these groups, and a total of 12 subjects were excluded from individual analyses. An additional 5 subjects were excluded because of inadequate sample collection. The study selection outline of the study participants, testing procedures, and subject selection is shown in **Figure 1**.

The clinical protocol was approved by the Institutional Review Board for each participating hospital, and for the New York City Department of Education. This study was conducted in conformity with the guiding principles for research involving humans (25). Written informed consent and assent were obtained from all parents and students, respectively. In the school setting, as the full test version of IVGTT is not practical, we used a modified version for the assessment of AIR and it has been validated in clinical trials to predict progression to the development of type 2 diabetes (26–28). In this study, the GDI was calculated as log_10_(AIR × fasting glucose concentration/fasting insulin concentration). The use of this index was modified based on our own experience and published in a previous work (24).

### School Visit

During the school visit, each consented subject underwent measurement of height and weight (using the study scale and stadiometer), blood pressure, and waist circumference (measured just above the iliac crest). Body mass index (BMI) was calculated as weight (kg)/height (m^2^), and age- and gender-appropriate BMI z-scores were calculated based on the 2000 Centers for Disease Control and Prevention reference data (29). Detailed medical and family histories were taken from subjects and their respective parents for chronic conditions such as obesity, diabetes mellitus, and hypertension. Body composition was analyzed by bioelectrical impedance (Body Fat Analyzer, Model HBF-306, Omron, Gays Mills, WI). Blood tests relevant to glucose homeostasis (fasting blood glucose and insulin), dyslipidemia [total serum cholesterol, triglyceride (TG), high-density lipoprotein-cholesterol (HDL-C), low-density lipoprotein-cholesterol (LDL-C)], inflammation TNF-α, adiponectin, CRP, IL-6, retinol binding protein 4 (RBP4)] and IGFBP-1 were drawn from fasted subjects between 8 and 10 a.m. Following baseline blood samples, each subject was then given 0.5 gm kg^-1^ of glucose (25% dextrose, maximum 25 g) infused intravenously over 3 min *via* the indwelling butterfly needle that was used to obtain fasting lab values. After flushing the line with saline, blood was drawn through the same indwelling line for measurement of serum insulin concentration at 1, 3, and 5 min after glucose administration as part of IVGTT. After completion of testing, subjects were given breakfast and escorted back to their usual classes. Pubertal or Tanner staging was not performed due to the school setting of the study, and this was a major limitation of the study.

### Laboratory Analyses

IGFBP-1 was measured by ELISA (Beckman Coulter, Brea, CA) with an intra-assay precision of 2.5% and an inter-assay precision of 3.8%. RBP4 was measured by ELISA (ALPCO, Salem, NH). Glucose was measured by the hexokinase method (Glucose/HK; Roche Molecular Biochemicals, Werk Penzberg, Federal Republic of Germany). Plasma insulin was measured by solid-phase 125-I-RIA (Coat-A-Count; DPC, Los Angeles, CA). Total cholesterol, HDL-cholesterol, and triglycerides (TG) were determined by an autoanalyzer (Integra 400 Plus, Roche Diagnostics, Indianapolis, IN). LDL-Direct was calculated from total cholesterol, HDL, and TG using the Friedewald formula. Adiponectin was measured by RIA (Millipore, Billerica, MN). IL-6 and TNF-α were measured by ELISA (R&D Systems, Minneapolis, MN). CRP was measured by turbidimetric assay (Roche Diagnostics, Indianapolis, IN). The assays for IGFBP1 and TNF-α were performed in batches.

### Statistical analysis and Calculations

The data are presented as mean ± SD unless otherwise indicated. Initial comparisons were made between groups by ANOVA. Insulin sensitivity was calculated as QUICKI (1/log fasting insulin + 1/log fasting glucose (mg/dl)) and HOMA (fasting glucose (mg/dl) × fasting insulin/405). Islet cell function was measured as AIR = (mean insulin rise 3–5 min after IV dextrose) and GDI = [log_10_ (AIR × [fasting glucose]/[fasting insulin])]. GDI was calculated in 75 of 98 subjects (incomplete data sets beyond fasting values). GDI is a means of assessing pancreatic islet cell capacity to rapidly secrete insulin that essentially “corrects” the AIR for inter-subject differences in insulin sensitivity (24). Correlations were calculated among IGFBP-1 and other clinical and biochemical variants using Pearson’s correlation coefficient. Multiple forward stepwise linear regression analyses were performed to determine which dependent and independent variables contributed independently and significantly to the adiposity-related comorbidity risk factors. Normality of distribution was ascertained by Wilk–Shapiro testing. Statistical significance was prospectively defined as p_α_ <0.05. The ROC curve for IGFBP-1 was constructed from the data using fasting insulin of <15 as insulin-sensitive and ≥15 as insulin-resistant children.

## Results

The mean BMI of the study group was 22.7 ± 4.9 kg/m^2^ (range: 13.8–36.8 kg/m^2^). Eighteen students (18%, 6 females, 12 males) had BMI more than 2 SDS for age and sex (**Table 1**). Total cholesterol (172 ± 34, vs. 159 ± 27, p < 0.05) and adiponectin (13.4 ± 4.8 vs. 11.1 ± 4.6, p < 0.01) were significantly higher in males. CRP was significantly higher in females (4.8 ± 5.8 vs. 2.8 ± 3.4 ng/ml, p < 0.05).

**Table 1:** Comparison between different groups.

Variable	BMI Z-score < 1 SDS N = 64	BMI Z-score 1-2 SDS N = 16	BMI Z-score > 2 SDS N = 18	All (n = 98)
Subjects	36 males, 28 females	9 males, 7 females	12 males, 6 females	57 males, 41 females
Age (years)	12.5 ± 0.97	12.7 ± 1.0	12.5 ± 0.85	12.5 ± 0.96
BMI (kg/m^2^)	19.8 ± 2.3 *^b^ ^c^*	25.8 ± 0.9 *^b^ ^d^*	30.7 ± 2.9 *^c^ ^d^*	22.7 ± 4.9
BMI z-score	0.09 ± 0.5 *^b^ ^c^*	1.56 ± 0.2 *^b^ ^d^*	2.8 ± 0.8 *^c^ ^d^*	0.84 ± 1.2
Body fat %	23.8 ± 6.4 *^b^ ^c^*	31.8 ± 4.6 *^b^ ^d^*	37.5 ± 3.5 *^c^ ^d^*	27.6 ± 7.9
Waist (cm)	69.6 ± 10.2 *^b^ ^c^*	86.7 ± 7.5 *^b^*	91.5 ± 12.6 *^c^*	76.4 ± 13.9
IGFBP1 (ng/mL)	27.3 ± 14.3 *^b^ ^c^*	13.9 ± 8.9 *^b^*	9.3 ± 9.4 *^c^*	21.8 ± 14.9
Glucose (mg/dL)	92.8 ± 7.1	92.9 ± 5.3	92.3 ± 6.7	92.7 ± 6.7
Insulin (µIU/mL)	7.9 ± 5.7 *^b^ ^c^*	14 ± 8.8 *^b^*	14.4 ± 7.6 *^c^*	10 ± 7.2
AIR	65.3 ± 32.3 *^b^ ^c^*	135.7 ± 87.5 *^b^*	161.5 ± 119 *^c^*	92.7 ± 75
QUICKI	0.36 ± 0.02 *^b^ ^c^*	0.33 ± 0.03 *^b^*	0.33 ± 0.02 *^c^*	0.34 ± 0.03
HOMA	1.89 ± 1.68 *^b^ ^c^*	3.6 ± 2.6 *^b^*	3.9 ± 2.4 *^c^*	2.56 ± 2.18
GDI *^a^*	2.87 ± 0.32	2.87 ± 0.27	2.9 ± 0.25	2.88 ± 0.3
Cholesterol (mg/dL)	161 ± 31	169 ± 29	172 ± 33	165 ± 31
TG (mg/dL)	67 ± 32 *^c^*	78 ± 21	104 ± 56 *^c^*	76 ± 38
HDL (mg/dL)	56 ± 11 *^c^ ^d^*	51 ± 10 *^d^*	43 ± 8.7 *^c^ ^d^*	53 ± 12
LDL (mg/dL)	91 ± 25 *^c^*	102 ± 25	108 ± 28 *^c^*	96 ± 27
Chol/HDL	2.9 ± 0.7 *^b^ ^c^*	3.4 ± 0.6 *^b^ ^d^*	4.0 ± 0.9 *^c^ ^d^*	3.2 ± 0.8
TG/HDL	1.3 ± 0.9 *^b^ ^c^*	1.6 ± 0.6 *^b^ ^d^*	2.5 ± 1.5 *^c^ ^d^*	1.56 ± 1.1
Adiponectin (ng/mL)	13.6 ± 5.1 *^c^*	11.3 ± 3.9	9.5 ± 3.5 *^c^*	12.5 ± 4.9 *^c^ ^d^*
RBP4 (ng/mL)	19.9 ± 4.4	20.4 ± 3.4	21.4 ± 4.8	20.3 ± 4.3
TNF-α (pg/mL)	1.9 ± 1.37	2.05 ± 1.9	2.7 ± 2.1	2.0 ± 1.6
CRP (ng/mL)	3.4 ± 6.5 *^c^*	5.3 ± 6.1	6.5 ± 4.8 *^c^*	4.3 ± 6.2
IL-6 (pg/mL)	1.0 ± 0.7	1.16 ± 0.65	1.18 ± 0.67	1.06 ± 0.7

Groups were divided by BMI Z-score.

a Test completed in only 75/96 subjects.

b p-value <0.05 (between BMI z-score <1 SDS and BMI z-score 1 to 2 SDS groups).

c p-value <0.05 (between BMI z-score <1 SDS and BMI z-score >2 SDS groups).

d P-value <0.05 (between BMI z-score >2 SDS and BMI z-score 1 to 2 SDS groups).

**Figure 2:**
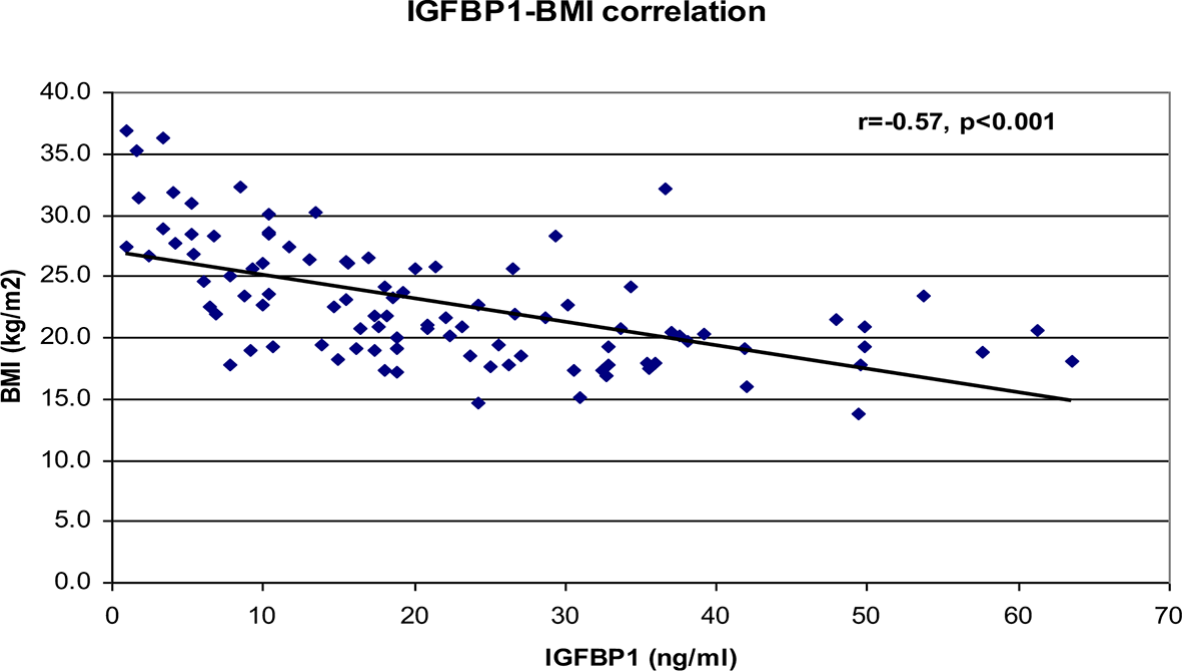
Inverse correlation analysis between IGFBP-1 levels and BMI (kg/m^2^).

**Figure 3:**
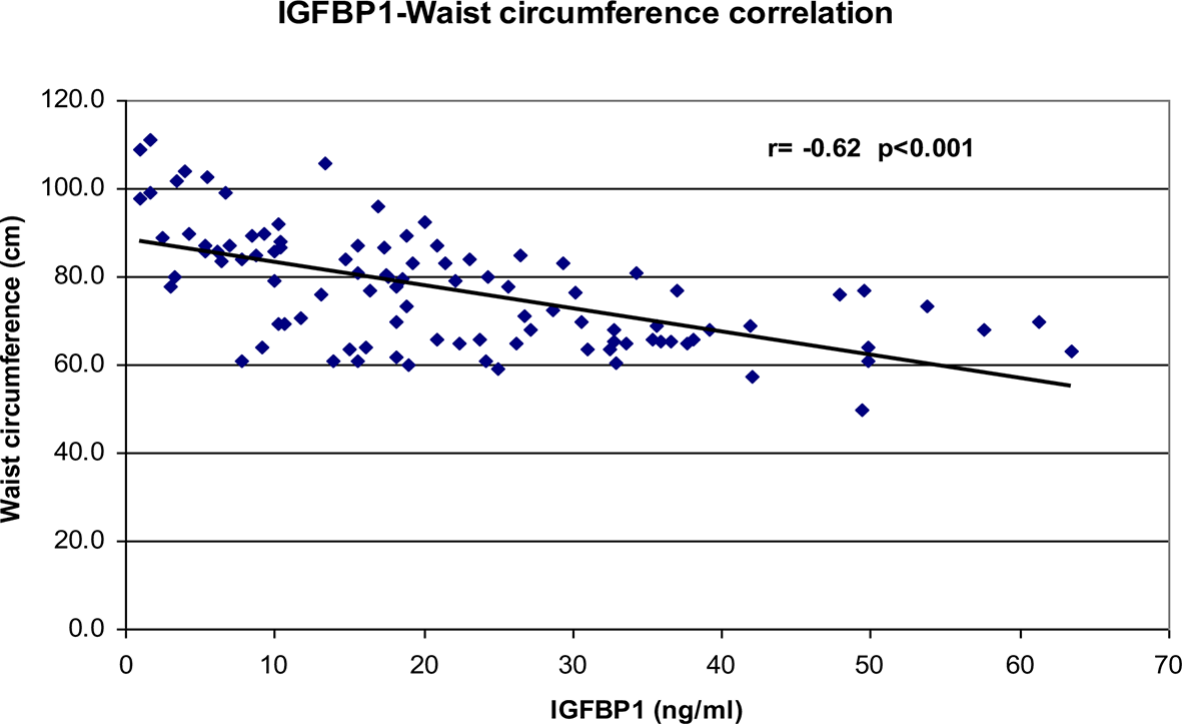
Inverse correlation analysis between IGFBP-1 levels and waist circumference (cms).

**Figure 4:**
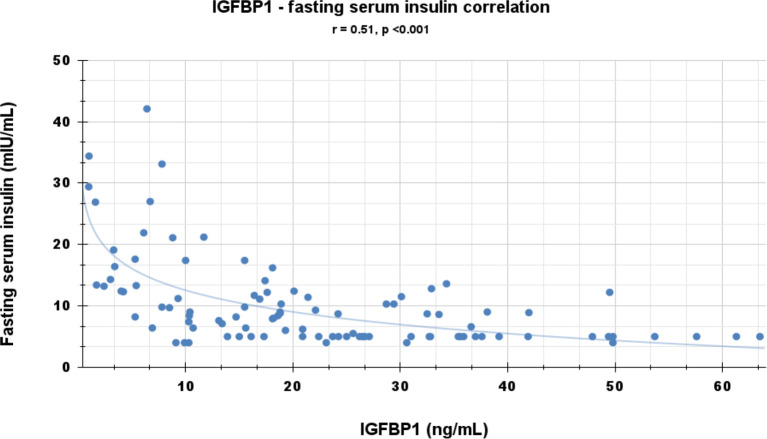
Inverse correlation of IGFBP-1 with fasting insulin levels.

**Figure 5:**
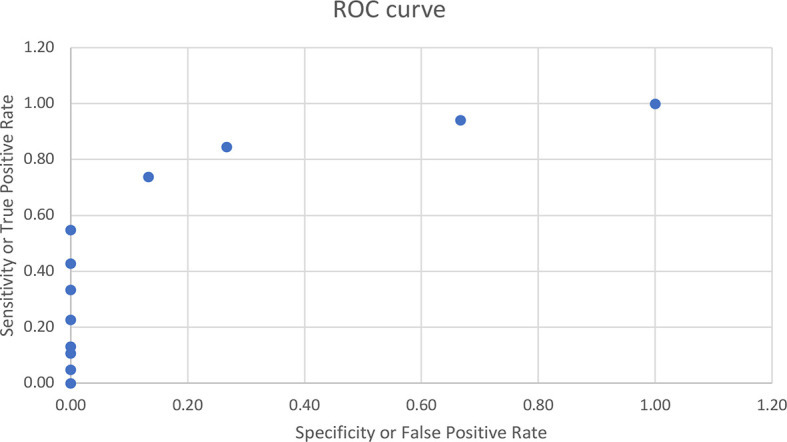
ROC curve for IGFBP-1 with the AUC of 0.92. The cutoff value of IGFBP-1 levels of <5 had sensitivity of 94% and specificity of 67% to identify insulin resistance in children.

Correlational analyses were performed relating circulating concentrations of IGFBP-1, anthropometric data, and indices of insulin sensitivity and dyslipidemia (**Table 2**). Circulating concentrations of IGFBP-1 were significantly and inversely correlated with age and all indices of body fatness such as body fat %, BMI (shown in **Figure 2**), and waist circumference (shown in **Figure 3**). IGFBP-1 correlated more strongly with waist circumference (**Table 2**, r = -0.62; p-value <0.001). Inverse correlation was seen with fasting insulin (shown in **Figure 4**), AIR, HOMA, TG, LDL, Chol/HDL, and TG/HDL ratios. In addition, circulating IGFBP1 concentrations were positively correlated with adiponectin, QUICKI, and HDL cholesterol (**Table 2**). The ROC curve for IGFBP-1 was constructed, and the AUC was high at 0.92. The cutoff value of IGFBP-1 levels of <5 had a sensitivity of 94% and a specificity of 67% to identify insulin resistance in children (**Figure 5**). There were no differences in the fasting insulin and IGFBP1 levels between the various racial/ethnic groups.

**Table 2:** Correlation analysis between IGFBP-1 and metabolic variables.

Variable	Correlation factor (r)	p-value
Age	-0.30	<0.005
BMI	-0.59	<0.001
BMI z-score	-0.33	<0.001
Body fat %	-0.42	<0.001
Waist circumference (cm)	-0.62	<0.001
Fasting insulin	-0.51	<0.001
AIR	-0.39	<0.05
AIR corrected for fasting insulin	-0.02	Not significant
QUICKI	0.54	<0.001
HOMA	-0.51	<0.001
Total cholesterol	-0.0016	Not significant
TG	-0.22	<0.05
HDL	0.34	<0.001
LDL	-0.1	Not significant
Chol/HDL	-0.30	<0.005
TG/HDL	-0.26	<0.01
Adiponectin	0.28	<0.005

### Subject Stratification

The subjects were stratified into 3 groups based upon the BMI Z-score. Out of all the subjects, 64 from 98 (65.3%) were in the group with a BMI Z-score <1 SDS, 16 (16.3%) subjects were in the group with a BMI Z-score 1 to 2 SDS, and 18 (18.4%) subjects were in the group with a BMI Z-score more than 2-SDS (**Table 1**). There were no differences between groups in age, IL-6, TNF-α, RBP4, fasting glucose, total cholesterol, and GDI.

The group with a BMI Z-score more than 2 SDS was compared to the group with BMI <1 SDS. The group with a BMI Z-score more than 2 SDS had significantly increased waist circumference, body fat, increased level of fasting insulin, AIR, HOMA, TG, LDL, Chol/HDL, TG/HDL ratios, CRP, low adiponectin, HDL, QUICKI, and low IGFBP-1 compared to the group with BMI <1 SDS.

The group with a BMI Z-score more than 2 SDS was compared to the group with a BMI Z-score 1 to 2 SDS. The group with a BMI Z-score more than 2 SDS had more body fat and Chol/HDL TG/HDL ratios and lower HDL compared to the group with a BMI Z-score 1 to 2 SDS. IGFBP-1 was lower at 9.3 ± 9.4 ng/ml, while in the group with a BMI Z-score 1 to 2 SDS, IGFBP1 was 13.9 ± 8.9, but it did not achieve significance with p = 0.14. There were no differences between groups with more than 2 SDS and 1 to 2 SDS in waist circumference, insulin, AIR, HOMA, QUICKI, TG, HDL, adiponectin, and CRP.

The group with a BMI Z-score 1 to 2 SDS was compared to the group with BMI less than 1 SDS. The group with BMI 1 to 2 SDS had decreased levels of IGFBP-1, QUICKI, increased waist circumference, body fat %, HOMA, AIR, fasting insulin, CHOL/HDL, and TG/HDL ratio compared to the group with a BMI Z-score less than 1 SDS. IGFBP-1 in the group with BMI less than 1 SDS was 27.3 ± 14.3 ng/ml (**Table 1**).

Based on these correlations of circulating concentrations of IGFBP-1 with multiple metabolic indices, adiposity-related comorbidities, age, and body fatness, multivariate linear regression analyses were performed to determine whether IGFBP-1 was significantly correlated with adiposity-related risk factors independent of body fatness. The comorbidity risk factor was treated as the dependent variable, and IGFBP-1 and BMI z-scores were treated as independent variables (**Table 3**). The r value for IGFBP1 and various dependent variables in **Table 3** is lower than that shown in **Table 2** because it has been adjusted for the effects of body fatness. There were independent effects of IGFBP-1 and body fatness on all variables except for triglycerides. Of note, semi-partial r values revealed that IGFBP-1 accounts for a greater fraction of the variance than body fatness for all indices of insulin sensitivity but not for indices of dyslipidemia risk, except for HDL cholesterol. To examine whether the intergroup differences in AIR and the significant correlation of AIR with body fatness and IGFBP-1 were reflective of insulin production or insulin sensitivity, the correlation of IGFBP-1 with AIR was calculated with and without fasting insulin levels as a covariate. No significant relationship between circulating concentrations of IGFBP-1 and AIR was found, once corrected for fasting insulin (**Table 3**).

**Table 3:** Correlation of dependent variables with IGFBP-1 and BMI z-score as independent variables.

Dependent variable	Semi-partial r		Overall adjusted r^2^
	IGFBP-1	BMI z-score	
Fasting insulin	-0.41 p < 0.001	0.20, p < 0.05	0.28, p < 0.001
QUICKI	0.43, p < 0.001	-0.24, p < 0.05	0.32, p < 0.001
HOMA	-0.41, p < 0.005	0.21, p < 0.05	0.28, p < 0.001
AIR	-0.29, p < 005	0.32, p < 0.005	0.22 p < 0.001
AIR (fasting insulin included)	-0.01, N.S.	0.19, p < 0.05.	0.32, p < 0.001
Adiponectin	0.20, p < 0.05	-0.25, p < 0.01	0.13, p < 0.001
TG	0.16, N.S.	-016, N.S.	0.06, p < 0.05
HDL cholesterol	0.25, p < 0.01	-0.21, p < 0.05	0.14, p < 0.01
Chol/HDL	-0.20, p < 0.05	0.26, p < 0.01	0.15, p < 0.01
TG/HDL	-0.19, p=0.07	0.21, p < 0.05	0.19, p < 0.005

The “Semi-partial r” refers to this adjusted correlation, and “adjusted r^2^” refers to a lowering of the r^2^ value because of including more variables. N.S., not significant.

## Discussion

Peripheral serum insulin levels are not a true reflection of secreted insulin, as almost 50% of secreted insulin is degraded passing through the portal circulation (14). Elevated insulin levels in the portal system are the initial manifestation of insulin resistance, and later peripheral insulin levels increase. IGFBP-1 levels are regulated via an insulin-response element in the IGFBP-1 promoter region, which explains the mechanism of insulin inhibition of IGFBP1 expression. Portal insulin directly inhibits the transcription of hepatic IGFBP-1 (18, 30, 31). In experiments, insulin infusion results in a suppression of plasma IGFBP-1 concentrations (32). Infants and children with hypoglycemia due to congenital hyperinsulinism have significantly lower IGFBP-1 (33). Low fasting serum IGFBP-1 levels reflect portal hyperinsulinemia even before peripheral hyperinsulinemia is detected (14). IGFBP-1 is higher in individuals with type 1 diabetes (hypoinsulinemic) (34), decreased in those with type 2 diabetes (hyperinsulinemic), and significantly diminished by insulin administration (35). In type 1 diabetes with insulinopenia, the IGFBP-1 levels are high because of low levels of portal insulin. The data in our study have confirmed a negative correlation between fasting IGFBP-1 and fasting serum insulin (r = -0.51; **Table 2** and **Figure 4**) and AIR (r = -0.39; **Table 1**). Travers et al. showed that insulin sensitivity derived from rapidly sampled IVGTT correlated strongly with IGFBP-1 levels in children (13). In our study, new findings were that IGFBP-1 independent of BMI negatively correlated with AIR and HOMA and positively correlated with adiponectin and TG/HDL ratio (**Table 3** and **Figure 2**). Motaghedi et al. in their study of obese children also revealed that insulin sensitivity reported from rapidly sampled IVGTT data correlated more strongly with IGFBP-1 than other measures of insulin sensitivity (5). A low level of IGFBP-1 is a marker for metabolic syndrome risk in both adults and children (36, 37).

Toddlers who were small for gestational age at birth have an increased lifetime risk of development of type 2 diabetes, and they also have significantly lower IGFBP-1 levels even before manifesting insulin resistance (23). Reinehr et al. and others have reported significant positive correlations of IGFBP-1 with various indices of insulin sensitivity in children with obesity (36, 38–40). IGFBP-1 has also been negatively correlated with the slope of trajectories of body weight gain across a spectrum of adiposity in adults and children (41). Katz et al. reported positive significant correlations of IGFBP-1 with adiponectin even controlling for insulin sensitivity, fat mass, body fat, and waist circumference. In this study, the correlation of IGFBP-1 with waist circumference was stronger than that compared to BMI and percent body fat (**Table 2**). The results in our study are in agreement with other similar studies in children (13, 36).

The major finding of this study was that the circulating concentration of IGFBP-1 can identify the presence of insulin resistance in the group with BMI 1 to 2 SDS, even before the known markers of insulin resistance such as elevated TG and even before decreased HDL and adiponectin levels are identified. There were no differences between groups with more than 2 SDS and 1 to 2 SDS in waist circumference. This demonstrates that decreased IGFBP-1 in obese/overweight children is simply not a consequence of increased fatness/adiposity, but it may reflect other independent metabolic processes conveying higher risk for dyslipidemia and type 2 diabetes. IGFBP-1 in this cohort of school-aged subjects is potentially an independent risk factor for these comorbidity risks. There was no difference seen in insulin and IGFBP1 levels in the various racial groups, however. The unique aspect of this study is to report the relation of IGFBP-1 in school-age children from very diverse racial and ethnic backgrounds.

One of the limitations of the study was that pubertal exam in the subjects was not performed due to the nature of the setting and insulin sensitivity is known to decrease in the mid-puberty age group (42).

The TG/HDL ratio was first proposed by Reaven et al. as another marker of insulin resistance, supported by other studies (43, 44). Multiple studies have shown the inverse correlation of IGFBP-1 levels with a TG/HDL ratio in adults (2, 3, 45). A lifestyle interventional study in obese subjects also revealed a negative correlation between IGFBP-1 and serum TG levels (36). The TG/HDL ratio in the present study was significantly and inversely correlated with IGFBP-1 levels (r = -0.26, p < 0.01; **Table 2**). This reinforces the fact that IGFBP-1 can be used as a marker of insulin resistance in children at high risk.

Adiponectin, an adipocyte-derived cytokine, reduces levels of free fatty acids in blood and has been associated with improved lipid profiles, better glycemic control, and reduced inflammation in diabetic patients (46). Low levels of adiponectin play a role in the development of insulin resistance and subsequent type 2 diabetes (47). Adiponectin levels have been shown to have a significant positive correlation with IGFBP-1 in adult studies (48). The present study also showed a positive correlation between IGFBP-1 and adiponectin levels (**Table 2** r = 0.28, p < 0.005).

In summary, low IGFBP-1 can be detected during childhood with a high risk for the development of insulin resistance. This cross-sectional study revealed the role of IGFBP-1 as an independent marker of insulin resistance and adiposity in this cohort of multiethnic diverse groups of schoolchildren. Although this is only a cross-sectional pilot study, it is necessary to perform similar studies on a larger and prospective scale including lean and obese children and children with metabolic syndrome. Our data indicate that by measuring IGFBP-1, the practitioners will have a reliable marker of insulin resistance.

## Data Availability Statement

The raw data supporting the conclusions of this article will be made available by the authors, without undue reservation.

## Ethics Statement

The clinical protocol was approved by the Institutional Review Board for each participating hospital, and for the New York City Department of Education. Written informed consent to participate in this study was provided by the participants’ legal guardian/next of kin.

## Author Contributions

Authors AB and RG contributed equally to the preparation of this manuscript the same as the first author. Authors MR and ST contributed equally to the preparation of this manuscript as a last author. RG, AB, and ST were involved in clinical care of the participants and their follow-up. Authors SS, DC, SA, LA, BL, and WR were involved in editing and revision of the manuscript. AB, ST, MR, PS, RR, and IF did a critical analysis of the manuscript and its editing. All authors contributed to the article and approved the submitted version.

## Funding

The study was supported by AMDeC, the Starr Foundation, and NIH grant numbers UL1RR0023568 and UL1RR0024156.

## Conflict of Interest

The authors declare that the research was conducted in the absence of any commercial or financial relationships that could be construed as a potential conflict of interest.

The reviewer EG declared a shared affiliation with the author RR to the handling editor at the time of review.

## Publisher’s Note

All claims expressed in this article are solely those of the authors and do not necessarily represent those of their affiliated organizations, or those of the publisher, the editors and the reviewers. Any product that may be evaluated in this article, or claim that may be made by its manufacturer, is not guaranteed or endorsed by the publisher.

## Abbreviations

IGFBP-1, insulin-like growth factor binding protein-1; IVGTT, intravenous glucose tolerance tests; OGTT, oral glucose tolerance tests; QUICKI, quantitative insulin sensitivity check index; HOMA, homeostatic model assessment for insulin resistance; AIR, acute insulin response; GDI, glucose disposal index.
